# High-throughput isolation of cancer cells in spiral microchannel by changing the direction, magnitude and location of the maximum velocity

**DOI:** 10.1038/s41598-023-30275-x

**Published:** 2023-02-24

**Authors:** Vahid Omrani, Mohammad Zabetian Targhi, Fatemeh Rahbarizadeh, Reza Nosrati

**Affiliations:** 1 Department of Mechanical Engineering, Tarbiat Modares University, Tehran, Iran; 2 Department of Medical Sciences, Tarbiat Modares University, Tehran, Iran; 3 Department of Mechanical and Aerospace Engineering, Monash University, Melbourne, VIC 3006 Australia

**Keywords:** High-throughput screening, Isolation, separation and purification, Lab-on-a-chip, Sensors and probes, Biological techniques, Biophysics, Cancer

## Abstract

Circulating tumor cells (CTCs) are scarce cancer cells that rarely spread from primary or metastatic tumors inside the patient's bloodstream. Determining the genetic characteristics of these paranormal cells provides significant data to guide cancer staging and treatment. Cell focusing using microfluidic chips has been implemented as an effective method for enriching CTCs. The distinct equilibrium positions of particles with different diameters across the microchannel width in the simulation showed that it was possible to isolate and concentrate breast cancer cells (BCCs) from WBCs at a moderate Reynolds number. Therefore we demonstrate high throughput isolation of BCCs using a passive, size-based, label-free microfluidic method based on hydrodynamic forces by an unconventional (combination of long loops and U-turn) spiral microfluidic device for isolating both CTCs and WBCs with high efficiency and purity (more than 90%) at a flow rate about 1.7 mL/min, which has a high throughput compared to similar ones. At this golden flow rate, up to 92% of CTCs were separated from the cell suspension. Its rapid processing time, simplicity, and potential ability to collect CTCs from large volumes of patient blood allow the practical use of this method in many applications.

## Introduction

Cancer is recognized as the second leading cause of death in the world. It is estimated that the number of cancer-related deaths will reach 13 million by 2030. The World Health Organization (WHO) believes that at least 30 percent of these deaths can be prevented if patients are diagnosed and treated before cancer metastasis occurs. Cancer metastases occur after circulating tumor cells (CTCs) have spread to the peripheral bloodstream from primary or secondary tumor sites^1^. Primary tumors are unlikely to cause deaths, but metastatic cells eventually account for 90 percent of all deaths while 0.01 percent lead to metastasis and most CTCs die in the bloodstream^2^. Due to a mutation, primary tumors may have different genomic information compared to metastatic CTCs. Oncologists compared CTCs and primary tumors and found that CTCs were more informative than primary tumors. CTCs were discovered about half a century ago, but their importance in cancer biology has only recently become apparent. This delay is primarily attributed to the difficulty in isolating CTCs (which occur at a rate of ~ 1–100 in ~ 1000–5000 leukocytes in the blood of patients)^3–5^.

There is great motivation for an isolation technique that allows rapid and efficient separation of CTCs^6^. Common diagnostic strategies for primary tumors depend on the analysis of clinical symptoms and imaging techniques. These methods can be used when the tumor has reached a definite size, and cannot be used to detect the existence of the tumor in its early stages^7,8^. Because cancer cells derived from primary solid tumors are bigger than blood cells, researchers have changed their approaches from affinity-based technologies to size-based separation. Due to this change, they can identify cancer patients in the early stages more easily^9^. Microfluidic methods have been highlighted as efficient and mighty tools for high throughput cell focusing by size^10,11^. Microfluidic separations are classified into two categories depending on energy consumption. Passive methods mainly use hydrodynamic forces, while active methods require external forces or controller to separate cells^12^. Active methods provide more precise separation but have expensive and complex components and lower throughput; more time is needed for external forces to act on the particles and overcome the hydrodynamic forces^13,14^.

Unlike conventional microfluidics methods, where inertia is negligible due to a very low Reynolds number (Re ≪ 1), inertial microfluidics is in the range of moderate Reynolds numbers (1 < Re < 100). In this range, inertia and fluid viscosity are finite and produce interesting effects, including (i) inertial migration and (ii) secondary flow^15,16^. Inertial microfluidics in straight, serpentine, and especially spiral patterns, is one of the most attractive methods of size-based separation. Due to its high throughput, simplicity, and lower cost, inertial microfluidics is an up-and-coming candidate in a wide range of biomedical applications^17,18^. Seo et al. first performed particle separation using a spiral microchannel in 2006. Then in 2008 Papautsky et al. used this method to separate 1.9 um particles from 7.32 um particles^19^. In 2009, Dicarlo et al. showed that this separation is due to the balance between lift and drag forces in a curvilinear spiral microchannel^20^. Since 2010, many efforts have been made to increase the efficiency and throughput of these methods using simple, low-cost platforms^21,22^.

In 2012, Sun et al. designed a spiral microchannel with an S turn, at the end of the first phase. While microchannel had high efficiency and purity, it had some disadvantages, the most critical problem was a low separation rate, which was about 0.5 ml/min^23^. The separation rate for this type of spiral microchannel doubled in the following years. In 2017, Rossum et al. isolated cells with efficiency of 88% and a purity of 91% at a flow rate of more than 1 ml/min^24^. In 2014, Karabacak et al. used a serpentine array microchannel to linearize and separate blood components^25^. In 2015, Sprenger et al. made a special spiral microchannel and separated cells between 2 and 18 µm, the entire range of blood cells, with a high flow rate and accuracy^26^. In 2017, Sonmez et al. creatively integrated serpentine and spiral patterns, and separated cells with high throughput and efficiency^27^. In 2018, Ding et al. designed a device including two spiral channels and a winding serpentine channel to isolate circulating cancer cells^28^.

In 2017, Lin et al. designed a spiral microchip with long loops, sharp angles, and a particular geometric pattern and managed to separate all blood components with a purity and efficiency of over 90%^29^. In 2019, Kosar et al. and zhang et al. created microfluidic chips that could separate cancer cells accurately by making changes to the dimensions, curvature radius, and serpentine array pattern. The value of these groups’ work is their chips' simplicity and low cost, which are two of the important criteria in fabricating microfluidic chips^2,30^. In 2017, Kim et al. used a straight channel with a unique cross-section to isolate cells^31^. In 2021, Warkiani et al. used trapezoidal cross-sections with various geometric patterns for cell separation and introduced a new cross-section. In 2020, Bridle et al. investigated the deformation of the cells in the spiral microchannel in detail with the help of experimental methods. Generally, many efforts have been made to increase the efficiency and speed of experiments^32–34^.

This study aims to indicate the performance of an unconventional spiral microchannel for cancer cells separation with high efficiency and throughput. For this purpose, several patterns were simulated and investigated; finally, by combining the spiral and serpentine array patterns and simultaneously using their advantages, a novel pattern with a U-turn was designed to simultaneously leverage the benefits of inertial forces and secondary flows to achieve an efficient cell separation with high throughput. The large aspect ratio makes the maximum velocity move more easily, and having a high curvature ratio makes it easier to manipulate the secondary flow.

The microchannel has been designed considering all the criteria, such as preventing clogging, simplicity of chip fabrication and testing, high efficiency and purification, and high throughput. At the next stage, standard particles migration behavior were investigated, and the capability of a designed chip for separating CTCs based on their size and shape was demonstrated. Finally, mixed cultures of BCCs and WBCs were used as a model system, and these cells were successfully isolated with an efficiency of more than 92% at a golden flow rate of 1.7 ml/min, which is in high agreement with the simulation and experimental results. Results for real cancer cells show a drop of about 2–3% in the separation efficiency compared to rigid standard particles, which is related to the effect of elasto-inertial force.

## Materials and methods

### Theory

**Figure 1:**
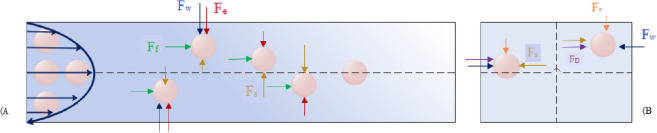
Dominant forces applied to particles within the microchannel at different transverse and longitudinal positions. (**A**) Microchannel length: The green arrows indicate the fluid drag force (F_f_), which moves the particles along the microchannel. The blue arrows indicate the wall induced lift force (F_w_), which pushes the particles toward the center of the microchannel. The brown arrows indicate the shear gradient lift force (F_s_) that pushes the particles toward the microchannel wall. (**B**) Microchannel cross section: The orange arrows indicate the rotating lift force (F_r_), which collects particles in the middle of the channel walls. Purple arrows indicate the Dean drag force (F_D_) that pushes particles from the inner wall to the outer wall. Finally, the red arrows indicate the elasto-inertial lift force (F_e_) that pushes the particles toward the channel's centerline.

In a straight microchannel, particles experience shear stresses that generate drag forces and normal stresses, creating lifting forces perpendicular to the flow direction^35^. In inertial microfluidics, the separation of particles depends on the balance between inertial lift forces and drag forces^36,37^. In Newtonian fluids, when particles are randomly released in the microchannel with a rectangular cross-section, particles follow system symmetry and focus on two equilibrium positions in the middle of the inner and outer walls^38,39^. However, in common applications of microfluidics, in a non-Newtonian fluid with a viscous and elastic behavior, particles deform and migrate to the center line of the channels. As depicted in Fig. 1, particles focus on specific lateral positions under the influence of six main forces in the Poiseuille flow^40^.

The first component, called the shear gradient lift force (F_S_), is due to the difference in magnitude of velocity between the fluid elements and creates the shear rate in each region. This shear gradient drives the particles to regions with higher shear rates. Therefore, particles close to the middle of the microchannel are propelled toward the walls of the channel^41^. However, if the particles get closed near the wall, they disrupt the streamlines. As a result, the pressure between particles and the microchannel walls increases^42^. The second component, called the wall-induced lift force (F_W_), is due to a pressure gradient near the microchannel wall that opposes the shear gradient lift force and pushes the particles back toward the center of the channel. Floating cells migrate to positions where these inertial lifting forces are in equilibrium, where the number of equilibrium positions is related to the channel cross-section^42^.

The third component is a smaller force called the rotational lift force (F_r_). This force is due to the rotation of the particles. As soon as the equilibrium locations of the particles are identified in a microchannel, rotational lift force dominates and gradually pulls the particles to the center point of all four channel walls^43,44^. When the diameter of the particles is small compared to the hydraulic diameter of the microchannel (particle confinement ratio (λ = a/D_h_, λ > 0.07)), the magnitude of the net inertial lift force (F_L_) is reported by Asmolov^45^:

1 $$F_{L} = \frac{{\upmu ^{2} }}{\uprho }{\text{Re}}_{p}^{2} C_{L} ({\text{Re}}_{c} ,x_{c} ),$$

2 $$F_{L} =\uprho a^{4} \left( {\frac{{U_{m} }}{{D_{h} }}} \right)^{2} C_{L} ({\text{Re}}_{c} ,x_{c} ),$$

where Re_c_ and Re_p_ (Re_p_ = Re_c_ λ^2^ > 1) are the Reynolds number of the microchannel and particle, respectively (It is accepted that Inertial focusing depends on dimensionless parameters such as λ, Re_c_, and Re_p_). ρ is the density of the fluid, μ is the fluid viscosity, and C_L_ (Re_c_, x_c_) is the lift coefficient of the net inertial lift force that is an indefinite function of the normalized particles' position at the microchannel cross-section, and the channel Reynolds number. This coefficient can be obtained from numerical simulation or experimental measurements, but in typical microfluidic applications, its value can be assumed to be 0.5. Shear gradient lift force dominates on the particles near the center line of the channel, While beyond ~ 2.0 D_H_ from the center of the microchannel, The sign of the lift coefficient changes, which indicates the superiority of the wall induced lift force over the shear gradient lift force^20,36,43,46^.

Introducing curvature to a straight microchannel creates a secondary flow that pushes the particles from the inner wall toward the outer wall of the microchannel because of the centrifugal effects. This secondary flow reverses through areas near the upper and lower walls of the microchannel. Thus, two reciprocating vortices are created in a curvilinear microchannel called Dean vortices. The Dean flow adds a new force on particles called the dean drag force (F_D_) that can be useful for manipulating the particles focusing positions in the microchannel. The balance between the F_D_ and F_L_ results in a size-based particle sorting mechanism across the microchannel. The velocity of this secondary flow can be estimated as below, where U_m_ = 3/2 U_avg_ is the maximum velocity of the microchannel, a is the particle diameter, D_h_ is the hydraulic diameter, and r is the curvature of the channel^47–51^.

3 $$U_{D} = 1.8 \times 10^{ - 4} De^{1.63} ,$$

4 $$De = {\text{Re}} \sqrt {\frac{{D_{h} }}{2r}} ,$$

5 $$F_{D} = 3\upmu \pi aU_{D} ,$$

6 $$F_{D} \sim \frac{a}{r}(U_{m} D_{h} )^{2} .$$

### Simulation

The lift forces fixate particles at equilibrium positions, while the Dean drag force causes particles to migrate around the cross-section. A new equilibrium position can be estimated from the ratio of F_L_ to F_D_, where δ is the curvature ratio^29^. F_D_ can be equal to, greater, or less than F_L_ based on various parameters such as the velocity field and microchannel pattern. At very low flow rates, F_D_ and F_L_ are too small for particle focusing, and particles remain scattered in the channel. At very high flow rates, F_D_ dominates, forcing the particles to follow the Dean secondary flow, which mixes the particles instead of focusing them. Thus, a golden flow rate range F_L_/F_D_ = 1 forms only one sharp equilibrium position^36,42^.

7 $$\frac{{F_{L} }}{{F_{D} }} = \frac{{{\text{Re}}_{c}^{n} }}{\updelta }\left( {\frac{a}{{D_{h} }}} \right)^{3} \quad (n \prec 0),$$

8 $$\updelta = \frac{{D{}_{h}}}{2r}.$$

**Figure 2:**
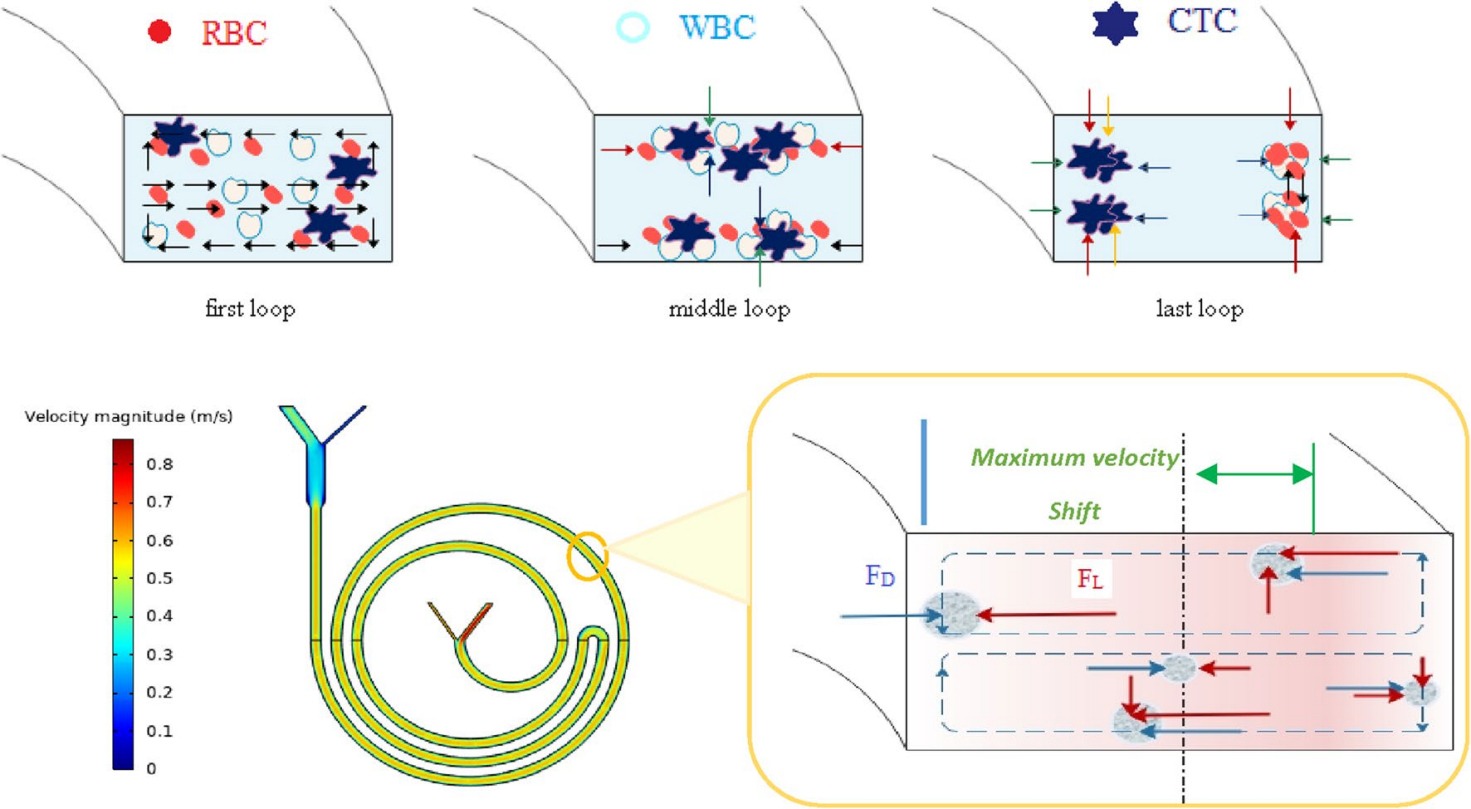
The effect of secondary flow on cell isolation in a curved channel, where larger particles accumulate near the inner wall and smaller particles near the outer wall. Firstly, the lift forces act on the particles and the particles accumulate in an equilibrium position in the middle of the walls. Finally, the smaller particles migrate toward the outer wall due to the presence of transverse secondary flows. The red arrows indicate the lift force, and the blue arrows indicate the drag force. The channel's curvature causes the maximum velocity to be transferred to the outer half of the channel. Larger particles are subjected to a larger lift force so they cannot pass through the outer half, and large particles initially in the outer half are driven into the inner wall by both lift and drag forces. On the other hand, the vertical lift force on small particles is not enough to send the particles up and down the channel to follow the flow, thus remaining in the outer wall.

For larger particles near the upper and lower walls of the channel, the inverse F_D_ is associated with the horizontal shear gradient to push the particles toward the inner wall. Suppose larger particles reach the area near the inner wall. In that case, they no longer follow Dean's vortices because larger particles experience more FL, forcing them to follow a streamline closer to the inner wall than smaller particles. The F_L_ confronts them and creates a force balance where larger particles can focus effectively. However, as shown in Fig. 2, F_L_ is weaker for smaller particles and cannot compete with F_D_. Thus, the smaller particles follow the Dean secondary flow until they arrive at the outer wall. In this region, due to the position of the particle, forces are weak and cannot circulate smaller particles, creating a focusing position for smaller particles. This difference in the focusing behavior of large and small particles, which mainly depends on the velocity field and curvature ratio, provides an opportunity to separate particles. Therefore, the dean flow helps to focus the particles in only one of the potential equilibrium positions at intermediate F_L_/F_D_^2,19,36,42,52,53^.

Considering the flow drag force (F_f_), and elasto-inertial force (F_e_), six forces should be considered to find the eventual position of the particles in the curvilinear microchannel. Elasto-inertial force mainly depends on Reynolds (Re) and Weissenberg (Wi) numbers. The ratio between these two dimensionless numbers corresponds to the elasticity number (El), which depends only on the channel's dimensions and the fluid's properties^40,54,55^. Various components of blood cells are deformable, and can be isolated in curvilinear microchannels based on their sizes. Since F_L_ is proportional to the fourth power of the particle diameter, it is more difficult for blood cells to focus in microfluidic channels due to their smaller size compared to CTCs. In order to move the smaller particles to the equilibrium position, more vital mixing forces are needed to achieve better focusing for these particles^2,56,57^.

In this study, a series of comprehensive studies using the λ, Re, and De have been developed. Results showed that as the velocity profile becomes smoother for wider channels, the shear gradient rate decreases in this direction, and the Dean drag dominates on the particles in the wider channels. The addition of curvature to the microchannel changes the shear gradient lift forces' direction and magnitude simultaneously through redistribution of the velocity profile. Therefore, a simple ratio of forces is insufficient for designing inertial focusing, and precise design of the curvature is required^46,58^. The main difference between this design and previous spiral microchannels is the U-turn located on the flow path^16^. This turn has a sharper curvature than the smoother curvature of the "loops" in conventional spiral channels; the combination of long loops and sharp turns increases the focusing of both the large cells (CTCs) and small cells (WBCs), while conventional spiral microchannels are less successful at focusing smaller cells. These features, along with a high flow rate and precise design of microchannel curves, increase focusing, which is clearly visible in the simulation results^2,29,59^.

In this study, COMSOL Multiphysics 6.0 was used to analyze the flow field in the microchannel and obtain cross-sectional velocity profiles. The flow was considered single-phase and incompressible, and the Navier–Stokes equations were solved using a laminar flow module. For boundary conditions, a constant flow rate was assumed at the inlet of the microchannel, and zero pressure was set at the microchannel outlet. Moreover no-slip, and bounce conditions were applied to the walls. Physics-controlled extremely fine mesh was used for constructing the 3D model. The complete mesh consists of about 500,000 domain elements with an approximate mesh quality of 0.9, and the GMRES solvent was selected among other default solvers. Lift and drag forces acting on particles were applied by coding and the particle tracing module. Finally, the particles were traced to the outlet in a curvilinear coordinate and observed that the simulated results agreed with the theory and explained the trends.

### Design and chip specifications

**Figure 3:**
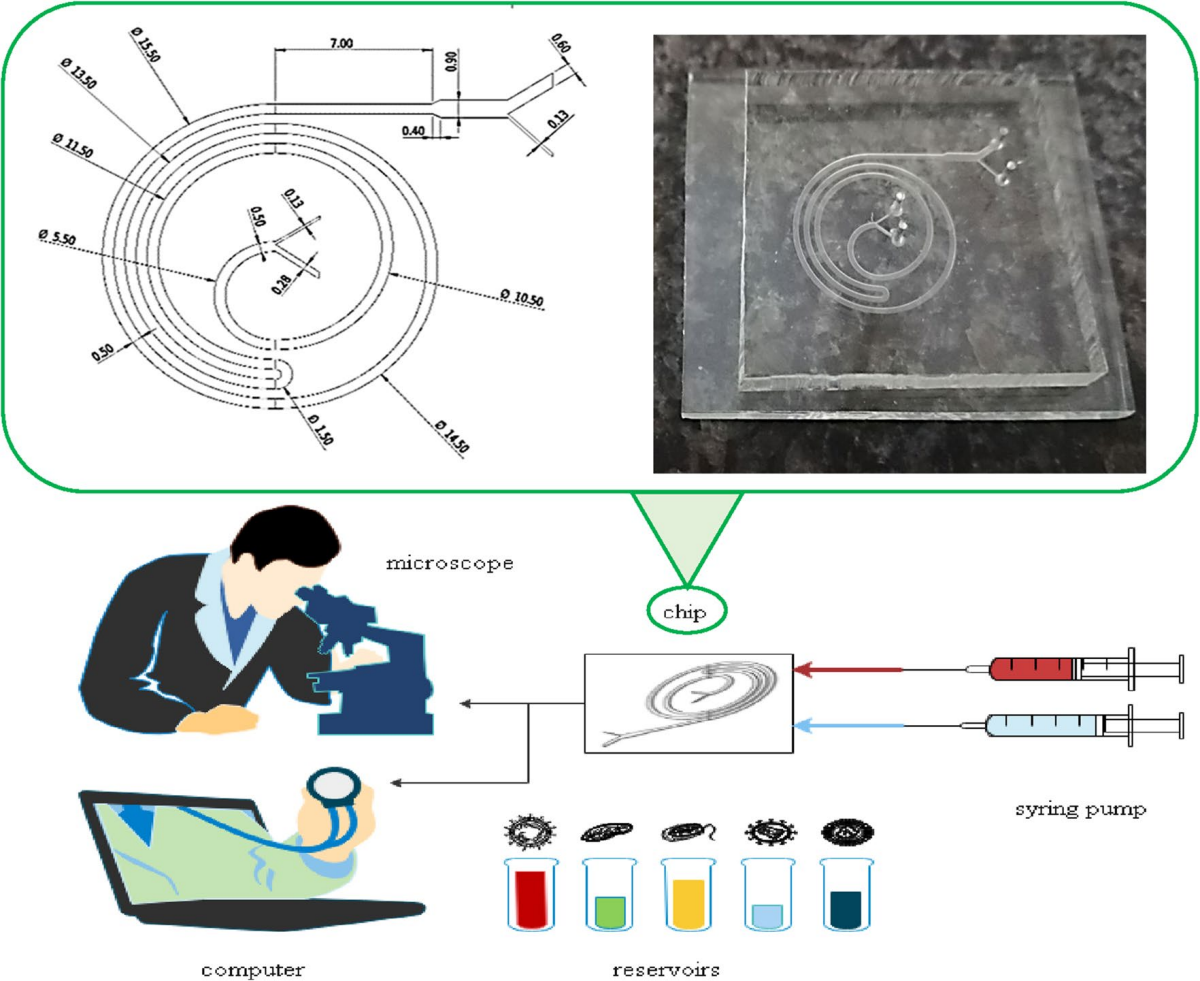
Microchannel geometric pattern with full details, and Schematic of the setup designed for experimental experiments.

The total length of the microchannel is 187 mm; this length can provide the necessary path for small and large particles to reach the maximum distance from each other in an equilibrium state. Further lengthening of the path can increase the possibility of channel clogging and particle deposition^60^. Its width is 500 µm and its height is 180 µm, large aspect ratio can make it easy to shift the maximum velocity in the microchannel cross-section and improve the ability to manipulate the particles^61^. The microchannel width increases from 500 to 900 µm before the flow is diverted to the outlets, and this extension makes it easier to design outputs and imaging them^62^. Curvature significantly affects focusing, so the microchannel pattern is mainly based on this feature. The microchannel consists of 4 loops and one U-turn to have a suitable curvature for separating BCCs from WBCs, and improves the focusing of smaller cells^29^. This microfluidic chip is made using a soft lithography method with PDMS and a glass substrate^63^. The schematic of the designed microchannel is shown in Fig. 3.

### Cell diameter

The lymphatic system is an extensive vascular network that can be considered the primary way to spread metastatic breast cancer cells (BCCs). The dynamics by which BCCs travel to distant sites in the lymph nodes has just been well understood. Particle tracking techniques were used to analyze the behavior of BCCs and standard solid particles of different diameters that were used to simulate cell flow in the lymph. Distinct differences between BCC and particle behavior indicate that morphology and size affect their response to lymphatic flow conditions^64^.

The BCCs adhered together and formed aggregate particles whose behavior was irregular. At the lymph flow rate, the MCF-7s were uniformly distributed across the channel compared to MDA-MB-231 cells that moved in the central region, indicating that metastatic MDA-MB-231 cells are subjected to a lower range of shear stresses in vivo. This suggests that size and deformability must be considered when modeling BCC behavior in the lymphatics. Human breast cell lines MDA-MB-231 (11–22 μm), MCF-7 (11–19 μm), and WBCs (6–16 μm), were used in this study^64,65^. Since about two-thirds of WBCs are in the range of 12–14 μm and about one-third of WBCs are in the range of 6–9 μm, the average diameter is assumed to be about 12 μm. Table 1 shows the characteristics of the cells used in this research; these cells were obtained from the Biotechnology Center of Tarbiat Modares University.

**Table 1:** Characteristics of cells floating in the lymphatic system.

Cell type	mcf-7 (μm)	mda-mb-231 (μm)	wbc (μm)
Min diameter	11	11	6
Max diameter	19	22	16
Average diameter	15	18	12

### Sample preparation

To evaluate the performance of the experimental system, 2–20 μm polydispersed microparticles (with an average diameter of 10 µm made of hollow glass) were selected for testing. These particle sizes were selected to match the cell size seen in the lymphatic in vivo. Hence monodispersed particles (with diameters of 5, 15.6 μm) are used to mimic the BCCs behavior. The particles were mixed at a 0.08% fraction in distilled water, with 1 vol. % of Tween-20 surfactant (Sigma-Aldrich, Dublin, Ireland) added to the solution to prevent particle aggregation. The dynamic viscosity of the distilled water and the lymph are 1 mPa s and in the range of 0.9–1.5 mPa s, respectively, and the densities of the distilled water and the particles are 1000 kg/m^3^ and in the range of 1050–1090 kg/m^3^, respectively. Due to the slight difference between them and distilled water, a percentage of glycerol was added to the particles Solutions^66^.

In order to prevent the effect of particles on the fluid or other particles (one-way coupling), the interaction of particles in the prepared suspension should be minimized to provide a homogeneous suspension with the appropriate concentration, assuming that the particle's size and distance are the same, the geometric location of the particles in the fluid is in triangular prisms whose sides are equal to L. The concentration of particles was considered L/D = 15 to avoid particle–particle interactions, where D is the diameter of the particle and L is the molecular mean free path^67^.

BCCs were suspended in PBS containing 3% FBS at a concentration of 1,000,000 cells per milliliter, and the cell lines were incubated for 20 min; the final number of cells was adjusted to 150,000 cells per ml by resuspending them in PBS containing 3% FBS using 0.05% trypsin and 1 mM EDTA. BCCs concentrations were chosen for data visualization and analysis purposes, mainly higher than the actual numbers reported in the literature, which are on a scale of 1–100 CTC per milliliter of blood^42,60,64^.

### Experimental setup

The sample suspensions were injected into the microchannel using a 10 mL plastic syringe by a syringe pump. Experiments were started with a minimum flow rate of 500 μL/min and continued up to the last flow rate of 2500 μL/min. Each experiment was repeated three times to check the tests' reproducibility. TYGON tubes (inner diameter: 250 μm, length: 15 cm) and fittings were used to connect the syringe tip to the chip. Before injecting the samples, the microchannel was washed with 70% ethanol and distilled water for aeration and sterilization. After that, PBS was pumped into the microchannel to prevent cell adhesion to the channel surfaces^60,68^.

The experiments were first performed with standard particles, and flow rate optimization was performed with various flow rates. The performance of this microfluidic chip for cell focusing can be expressed by counting cells/particles in each experiment. In the cell samples, the efficiency of CTCs separation is the ratio of the number of CTCs that came out of the desired outlet to the total number of cancer cells that came out of both outlets, this percentage is equal to the overall efficiency of the microfluidic device. Similarly, the purification shows what percentage of all the cells that have come out of the desired outlet for cancer cells are target cells.

Imaging was done both online and offline, online imaging was used to trace the particles and offline imaging was used to count particles. Offline imaging was done in the outlet, in such a way that the particles coming out of each outlet were collected in a separate container and then the suspension was homogenized and one milliliter of it was taken as a sample. Using a slide, five images was randomly taken from the sample and the number of particles was counted using ImageJ software and their average diameter was also calculated. For a better interpretation of the data, a suitable threshold was chosen for each image so that the debris does not affect the results and we do not lose any target cells. Figure 3 shows the schematic of the experiment setup^42,60^.

## Results

Based on simulation results, the buffer flow rate is equal to 1150 μL/min, and the sample flow rate is equal to 550 μL/min, which is significant Compared to similar ones. Tests using standard polydispersed (2–20 μm) and monodispersed (5, 15.6 μm) particles are in very high agreement with the simulation results; finally, the isolation of real cells (BCCs and WBC) was a confirmation of the excellent performance of the chip and its full compliance with theory, simulation and standard particles testing.

### Simulation results

#### The effect of microchannel curvature on the equilibrium position of particles

**Figure 4:**
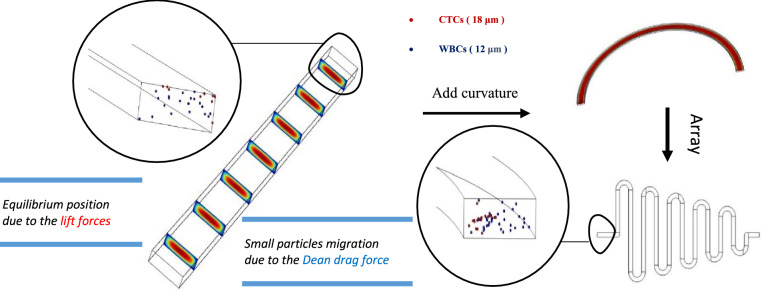
The results simulated in COMSOL software; at first, particles were traced in a straight microchannel, then curvature was gradually added to the microchannel and showed that due to the secondary dean flow, smaller particles began to migrate toward the outer wall. Finally, an array of these curved pieces was formed. Red particles are 18 µm, and blue particles are 12 µm in diameter. These particles mimic the behavior of WBCs and CTCs, respectively (COMSOL Multiphysics^®^ Version 6.0 is available: https://www.comsol.com/release/6.0).

In order to simulate as close as possible to the practical, 12, and 18 μm particles were used as mimicking of WBC and BCCs behavior. First, the separation of the particles was simulated in a straight microchannel. Then Curvature was gradually added to the channel, and it was observed that first the particles moved towards the inner wall. Then, due to the dean drag force, the smaller particles migrate to the outer wall. Finally, an array of these curved pieces was formed, and the particles were tracked from input to output. The results show that these microchannels have low efficiency, purification, and flow rates. The simulation results are shown in Table 2 and Fig. 4; each is interpreted and analyzed separately.

**Table 2:** Summary of simulation results along with schematic of their geometric pattern.

Num.	Channel	Pattern	Explain	Advantage	Disadvantage	Flow rate
1	Straight		Straight channel with 500 × 170 μm rectangular cross section	Simplicity	Very low efficiency Very low throughput	200 μL/min
2	Low curvature		Curvilinear channel with 500 × 170 μm rectangular cross section and low curvature	Simplicity	Very low efficiency Very low throughput	200 μL/min
3	High curvature		Curvilinear channel with 500 × 170 μm rectangular cross section and high curvature	Simplicity	Very low efficiency Very low throughput	200 μL/min
4	Serpentine		Serpentine channel with 500 × 170 μm rectangular cross section	Medium focusing Medium efficiency	Medium efficiency Low throughput	500 μL/min

#### The effect of microchannel pattern on the trajectory of the particles

Afterward, the particle trajectory had to be increased to complete the separation. For this purpose, the channel continues in a spiral mode, and the radius of curvature does not change significantly. Approximately constant curvature can be both an advantage and a disadvantage. As mentioned earlier, if this drag force exceeds a specific limit, it will cause the particles to mix, on the other hand, in conventional spirals, the efficiency is not very high. In serpentine microchannels, this problem is solved by sudden and arbitrary curvatures. The features of these two microchannels can be used simultaneously, and an unconventional spiral microchannel can be designed with an almost constant and long curvature path with desired curvatures. Therefore, U-shaped, L-shaped, and S-shaped turns can be embedded in the particle motion path.

To better understand the behavior of cells in a spiral microchannel, particle focusing in several conventional and unconventional spiral microchannels was simulated at different flow rates (500–2500 μL/min). The motion of particles with the sizes of 20 μm, 18 μm, 15 μm, 12, and 5 μm was investigated, and separation potential was examined in these microchannels. Through these simulations, the separation behavior of MCF-7 (11–18 μm), MDA-MB-231 (11–22 μm), and WBCs (6–16 μm) was investigated.

**Figure 5:**
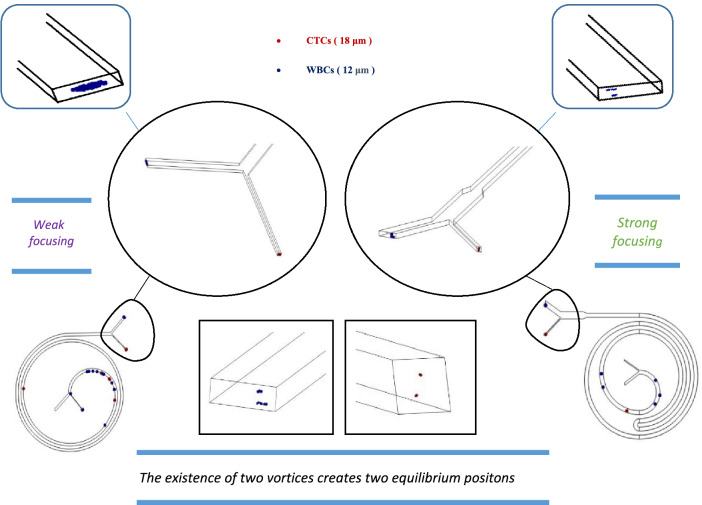
The COMSOL software simulation results, at first, particles were traced in a conventional microchannel at 0.9 mL/min, then a U turn was added to the way of particles and showed that due to the higher curvature ratio, smaller particles completely migrate toward the outer wall at 1.7 mL/min. The equilibrium position of the cells, which exist in two separate pieces at the outlet, indicates the presence of two transverse vertices. Red particles are 18 µm and blue particles are 12 µm in diameter. These particles mimick the behavior of WBCs and CTCs respectively (COMSOL Multiphysics^®^ Version 6.0 is available: https://www.comsol.com/release/6.0).

There are other essential factors to consider, for example, the required time to perform the test, the simplicity of performing and repeating the test, and the possibility of making the chip with standard tools. On the other hand, when the path is too long and the number of turns is more than a few, the possibility of channel clogging, particle deposition, and bubble formation increases. In this design, the chip included significant efficiency, an impressive flow rate, easy fabrication, and simplicity of testing at the same time. Table 3 and Fig. 5 summarize the simulation results. The equilibrium position of the cells, which exist in two separate pieces at the outlet, indicates the presence of two transverse vertices, which confirms the theory.

**Table 3:** Summary of simulation results along with schematic of their geometric pattern.

n	Channel	Pattern	Explain	Advantage	Disadvantage	Flow rate
1	Conventional spiral		Conventional spiral channel with 500 × 170 μm rectangular cross section	••Good focusing Approximately good efficiency	Approximately good efficiency medium throughput	850 μL/min
2	Unconventional spiral with one turn		Unconventional spiral channel with 500 × 170 μm rectangular cross section with one u turn	Very good focusing Very good efficiency Very high throughput	–	1600 μL/min
3	Unconventional spiral with two turns		Unconventional spiral channel with 500 × 170 μm rectangular cross section with two u turns	Good focusing Good efficiency Very high throughput	Design complexity Particle deposition Channel clogging	1800 μL/min
4	Unconventional spiral with three turns		Unconventional spiral channel with 500 × 170 μm rectangular cross section with three u turns	Good focusing Good efficiency Very high throughput	Design complexity Particle deposition Channel clogging	1800 μL/min
5	Unconventional spiral with multiple turns		Unconventional spiral channel with 500 × 170 μm rectangular cross section with multiple U, L, and S turns	Good focusing Good efficiency Very high throughput	Design complexity Particle deposition Channel clogging	2100 μL/min
6	Modified Unconventional spiral with one turn		Modified unconventional spiral channel with 500 × 170 μm rectangular cross section with one u turn and expansion in outlet	Very good focusing Very good efficiency Very high throughput	–	1700 μL/min

#### The effect of changes in the magnitude, direction, and location of the maximum velocity

In curvilinear microchannels with turns, sudden changes in curvature ratio can change the Dean flow direction and magnitude instead of being constant. Before interpreting the results, it is helpful to mention that three essential factors should be considered to analyze better the forces acting on the cells: The Dean drag force direction and magnitude, which depends on the vertical position of the cells in the microchannel, and the flow velocity maxima shift, that affects the magnitude of the shear gradient lift force. Because the velocity profile in straight microchannels is parabolic, maximum velocity is located in the middle of the microchannel. Adding curvature to the microchannel pattern causes not only the creation of the F_D_ but also changes the maximum velocity location, which depends on Re, curvature ratio, and cross-section. The direction and presence of the forces acting on cells depend on the cell's position in the microchannel.

**Figure 6:**
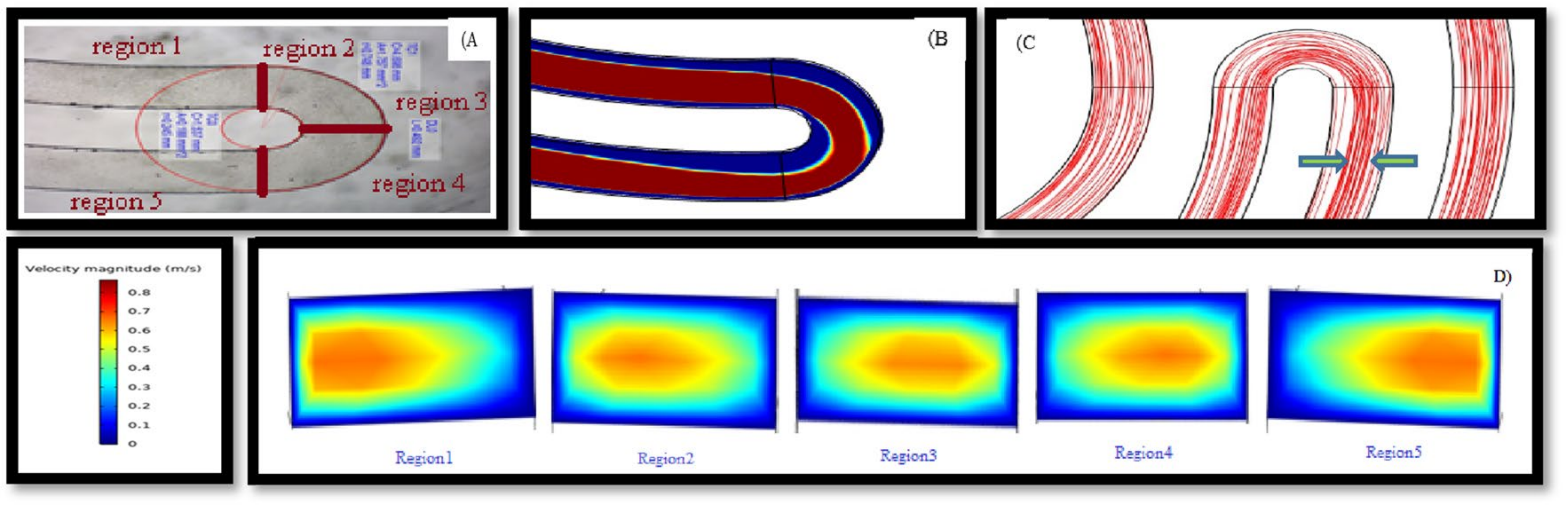
maximum velocity displacement due to high ratio turn. (**A**) Showed the turn of the microchannel which is made by soft lithography under the microscope. (**B**) Illustrated the velocity field as well as maximum velocity displacement along the microchannel. (**C**) This plot shows the streamlines of the flow field inside the microchannel, it is clear that the compression of these streamlines increases after the U-turn, and (**D**( indicated that maximum velocity had been shifted from outer wall into inner wall.

An image of the chip made by the soft lithography method is shown in Fig. 6, and the maximum velocity displacement due to the U-shaped turn is simulated. When smaller cells are in region 1, where the maximum velocity is in the microchannel's outer half, these cells are pushed toward the outer wall by the influence of F_D_. The balance of F_L_ and F_D_ results in forming a concentrated cell line near the centerline of the microchannel and inclined to the outer wall. However, this force cannot push bigger particles because lift force dominates in the larger particles. When cells arrive at region 2, since the maximum velocity is near the centerline of the cross-section, smaller particles that were close to the outer wall in the previous position start to move toward the inner wall by both F_L_ and F_D_. When the cells reach region 3, the maximum velocity is in the centerline like a straight microchannel, and enhanced F_D_ and F_L_ push the smaller particles toward the outer wall (the inner wall becomes the outer wall and vice versa).

When particles arrive in region 4, small particles can move close to the outer wall due to enhanced F_D_ and F_L_. In this region, the vertical and horizontal magnitude of F_S_ increased so that larger particles that already are in the outer wall from the previous position migrate to the top and bottom of the channel Because the lift force is directly related to the fourth power of the diameter, As shown in Fig. 2, the larger particles get closer to the top and bottom of the microchannel, and after that, larger particles start to migrate to the inner wall due to both enhanced F_D_ and F_L_. When cells arrive at region 5, larger particles still migrate to the inner wall and make an equilibrium line near the inner wall. On the other hand, smaller particles are in the equilibrium line near the other wall, so the distance between the smaller and larger particles increases significantly.

At low Re, smaller cells first focus in a broad band near the centerline due to relatively small inertial lift and Dean drag forces combined with the low flow velocity. The focusing is slightly improved with an increase in the Re or curvature. At first glance, this behavior of the cells is unexpected without a complete assessment because the F_S_ and F_D_ change with a_p_^3^ and a_p_, respectively. An increase in flow rate results in an increase in both F_S_ and F_D_. However, F_S_ increases more than F_D_ in larger particles leading to migration. This migration behavior is not clearly seen in smaller particles, because of their size. The horizontal and vertical components of F_S_ acting on smaller particles are smaller than larger particles at the same flow rate. Figure 6 shows the displacement of the maximum velocity in each of the five regions mentioned.

In the simulation, according to theory, 1000 white blood cells and 10 cancer cells were used to simulate the isolation process and the feasibility of separating these cells as close as possible to reality. In the experimental, to validate this simulation, firstly, standard particles with diameters of 2–20 µm were used. Although the separation of this range from cells is not the main goal, and in this research white blood cells and cancer cells are separated from each other, this range of particles diameter includes all the cells in the blood. The separation of this range was also investigated in the simulation, and it should be noted that if the diameter of the particles is closer to each other, it will be more difficult to separate them, so separating 12 and 18 μm particles can be a good choice, considering the average diameter of different blood components. Finally, real cell samples were isolated, and at a flow rate of 1.7 mL/min, cells were isolated with an efficiency and purity of over 90%, which is in very high agreement with the simulation results.

### Experimental results

#### Validation and feasibility of separation by standard particles

A mixture of monodisperse (5 and 15.6 μm) and polydispersed (2–20 μm) microparticles were tested for Validation and feasibility of separation. The particles remain dispersed in the microchannel until Q = 1 mL/min. At Q = 1.25 mL/min, particles roughly start to focus on the microchannel. The focusing line becomes narrower at 1.5 mL/min so that almost all particles are directed to the desired outlet. Then, by increasing the flow rate to Q = 1.7 mL/min, particles focus sharply, and almost all the particles exit from the desired outlet with approximately 94% focusing efficiency. At higher flow rates, particles begin to mix again, which causes a significant decrease in efficiency. Table 4 shows the details of the experimental tests with standard particles.

**Table 4:** Experimental test results for standard particles at various flow rate.

Test	Buffer (mL/h)	Sample (mL/h)	Outlet	Particle number	Efficiency %	Purity %
1	40	20	Outlet 1	51	60.7	70.1
			Outlet 2	121	55.3	60.3
2	48	24	Outlet 1	63	70.6	73.9
			Outlet 2	153	66.4	68.8
3	55	27	Outlet 1	78	84.2	86.6
			Outlet 2	195	82.3	88.7
4	60	30	Outlet 1	97	88.8	91.1
			Outlet 2	237	87.5	89.5
5	68	33	Outlet 1	113	94.1	95
			Outlet 2	272	93.2	94.8
6	80	40	Outlet 1	132	67.4	75.2
			Outlet 2	302	65.9	73.1

**Figure 7:**
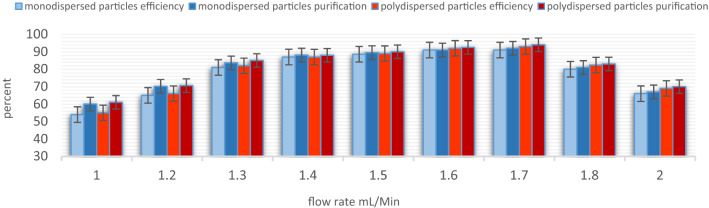
The bar chart for particles separation results at various flow rates.

**Figure 8:**
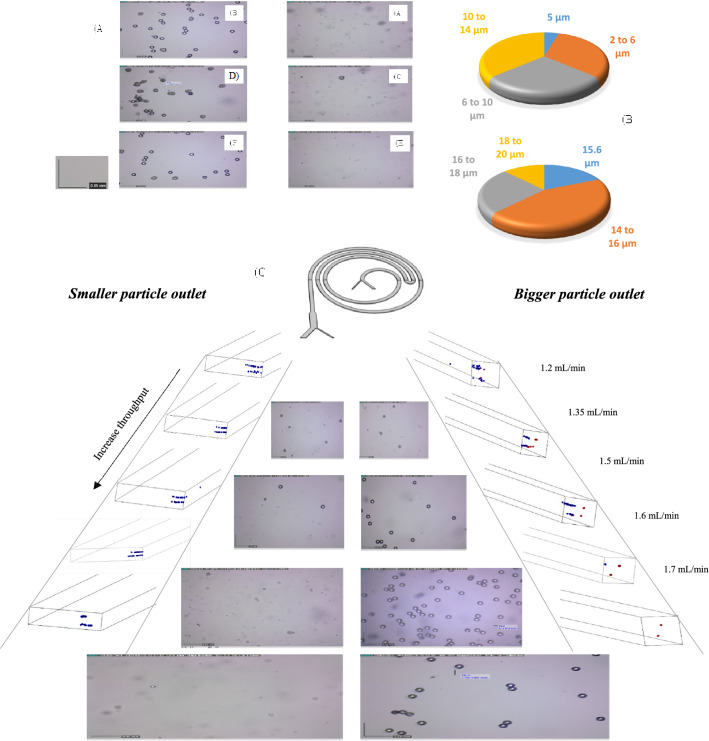
Simulation and experimental results for particles separation at different flow rates, In order to find the optimal flow rate, the flow rate is increased from 1 mL/min to 2 mL/min, the outlets are imaged and analyzed at each stage. (**A**) Experimental results for particles separation, (**A**) and (**B**) illustrated the desired outlets for smaller and bigger particles respectively at 1.4 mL/min. (**C**–**F**) Showed the same result at 1.55 mL/min and 1.7 respectively. The 1.7 mL/min result was better than other flow rates. (**B**) Indicate the size distributions of standard particles. (**C**) The optimum flow obtained by both simulation and experimental methods was approximately 1.7 mL/min. At this flow rate, more than 90% of smaller and bigger particles are separated.

For flow rates between Q = 1.4–1.7 mL/min, the efficiency of separation was more than 80%. When the flow rate increased from Q = 1.4 mL/min to 1.7 mL/min, the separation efficiency increased from 81 to 94%. An excessive increase in flow rate caused a significant decrease in efficiency so that only 65% of target particles were observed at the desired output at Q = 2 mL/min. Figure 7 shows the results of experimental testing at flow rates of 1.4, 1.55, and 1.7 mL/min. The behavior of the particles varied substantially with flow rate, as shown in Fig. 8, to optimize the separation, 12 and 18 μm particles were used for simulation, and polydispersed particles were used for experimental testing.

#### Isolation of breast cancer cells from white blood cells

To have a better Comprehension, since particle behavior significantly depends on size, our priority in analyzing the results relies on the size proximity between the cells and the particles. The size of the WBCs is between 6 and 16 μm, and their average diameter is 12 μm. The focusing trend of WBCs is more similar to the 10 μm particles. Additionally, the size distribution of MCF-7 is between 11 and 21 μm, and the average diameter of MCF-7 cells is 15 μm, which is closer to the 15.6 μm compared to 20 μm, and, the focusing behavior of the MCF-7 cells is similar to the 15.6 μm particles. The average diameter of the MDA-MB-231 cells is 18 μm between 15 and 20 μm; the separation trend of this cell is a combination of the 15.6 and 20 μm particles' trends.

**Figure 9:**
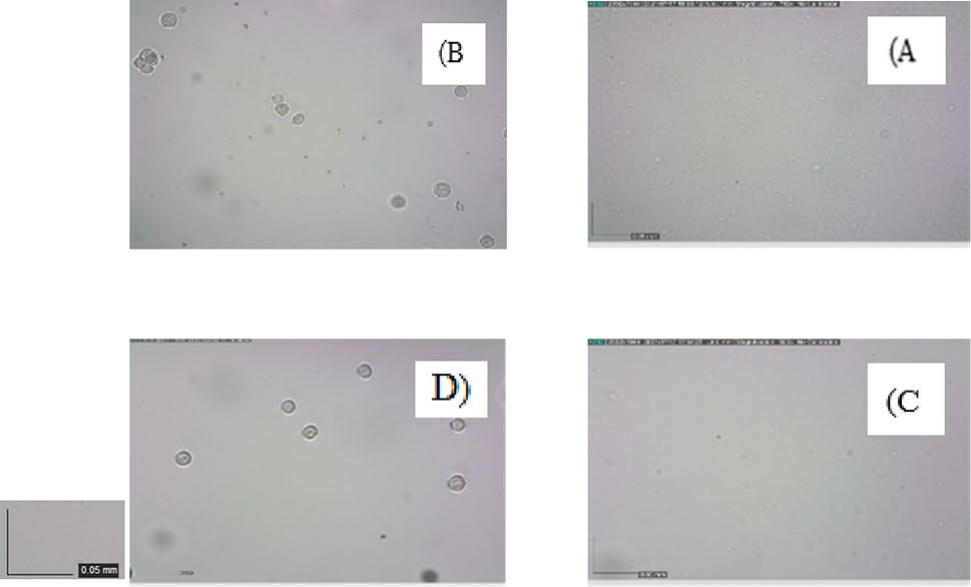
Experiment results for cells separation at golden flow rate. (**A**) and (**B**) illustrate the row samples of white and cancer cells without any pre-processing, respectively (**C**) illustrates the desired outlet for white blood cells (smaller cells) and (**D**) illustrates the desired outlet for cancer cells (bigger cells) at the 1.7 mL/min. In this flow rate, more than 90% of both CTCs and WBCs separate and purify.

**Figure 10:**
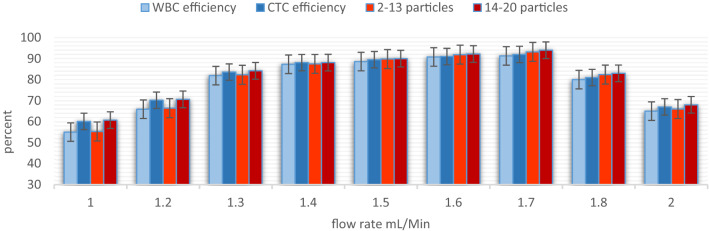
The comparison between real cells and rigid particles at various flow rate.

The highest efficiency occurs at Q = 1.7 mL/min, which Proves that standard particles can imitate the behavior of cells. Similar tests were conducted with real cells (mixture of WBC and BCCs) based on the golden flow rate obtained by the experiment and simulation, and the separation performance was quantified based on cell counts. Blood samples were processed with pre-processing and were lysed prior to injection into the microchip to prevent RBC interference. BCCs enumeration in these samples was considered much higher than the 2 CTC-like cells/mL were found in healthy bloodstreams. This experiment showed that the designed chip is useful to isolate cancer cells and more than 90 percent of cancer cells can be isolated from the patients' blood samples at a high throughput. Figures 9, 10, and Table 5 indicates the results of BCCs and WBC separation performance. Each experiment was repeated three times with a standard deviation of 0.2, indicating that the experiments have good reproducibility.

**Table 5:** Experimental test results for cell samples at optimum flow rate.

	Buffer (mL/h)	Sample (mL/h)	Outlet	Number of particles	Mean diameter (μm)	Efficiency %	Purity %
1	55	27	Outlet 1	71	14.2	88.1	89
			Outlet 2	173	9.9	87.3	87.2
2	60	30	Outlet 1	94	14.8	89.2	90.1
			Outlet 2	216	10.2	89.1	88.9
3	68	34	Outlet 1	107	15.3	91.9	92
			Outlet 2	258	10.7	91.3	91.6

In contrast to a rigid particle, due to the deformability of cells, elasto-inertial lift force act on cells, which may cause particles' lateral migration. Thus, the combined force on the cell is more than that act on a rigid sphere of the same volume in Poiseuille flow. It is worth noting that the focusing power was also increased significantly without any considerable loss of CTCs viability. Figure 10 compares the results for solid particles and deformable cells, which shows a slight drop of about 2–3% in the results for real cells, the reason for this reduction in efficiency is related to the elasto-inertial lift forces and deformation of the cells, which indicates that the deformation of the cells should also be considered in the simulation.

### Online particle tracing

**Figure 11:**
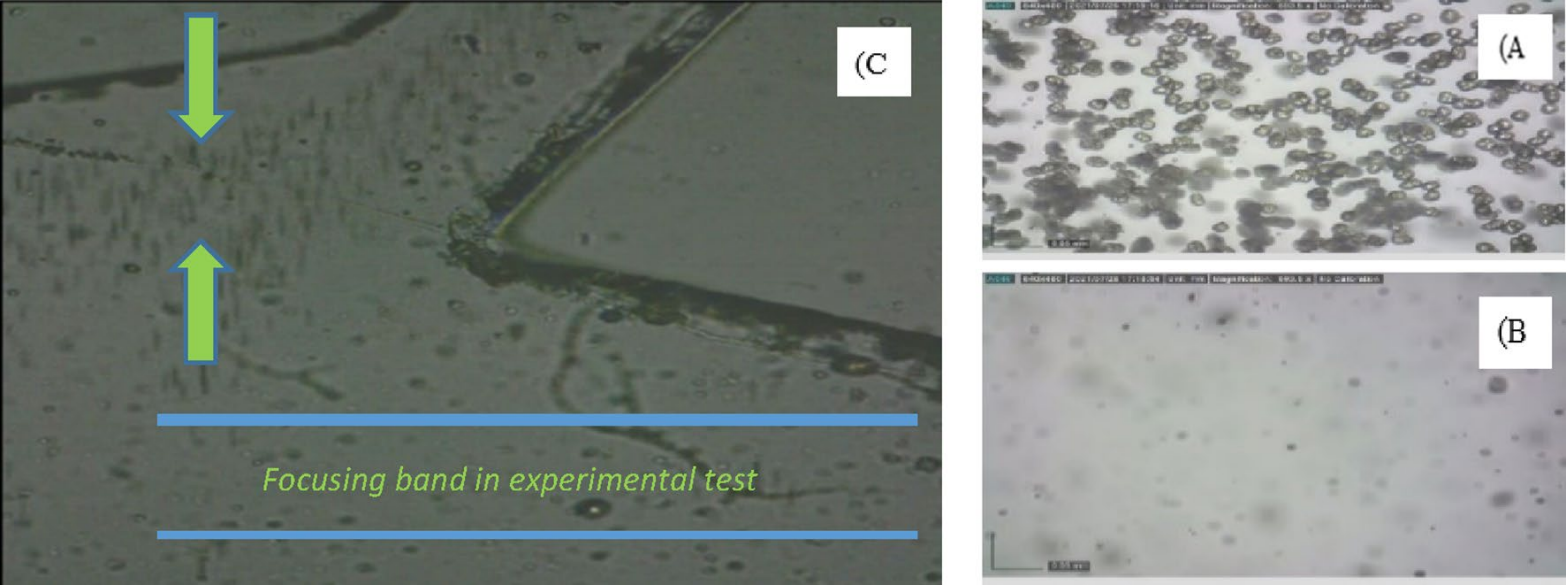
(**A**) and (**B)** show the outputs associated with larger and smaller particles, respectively. (**C**) Online trajectory of high concentration cell samples at golden flow rate.

The particle trajectories obtained from the processed videos of high-concentration suspension of rigid particles were in good agreement with the simulation results, as shown in Fig. 11*.* While in the simulation, the focusing bands were narrower than the experimental result. The fact that solid particles are not entirely spherical is the reason for this slight difference in the results. However, these results also show that Q = 1.7 mL/min is the golden flow rate to maximize the separation efficiency, which is in fair agreement with the simulation results.

## Discussion and conclusions

Due to recent advances in the physics of micro-flows, micro-particle motion, and genomic characterization tools, the study of tumor heterogeneity to better understand tumor progression and treatments is more accessible than ever by using the NGS method. Experts believe studying the genetics of CTCs isolated from the blood can be very important in diagnosing and treating the disease. This method can be used in future clinical studies using the genomic information of CTCs isolated by microfluidic chips as biomarkers to monitor solid cancer cell treatment.

To separate blood components from each other in a truly label-free method, we developed a strategy using inertial microfluidics that not only uses the delicate balance of inertial forces and Dean drag force, but also combines high curvature turn to change the direction of the fluid streamlines. The behavior of white blood cells with a mean diameter of 12, MDA-MB-231, and MCF-7 with a mean diameter of 18 and 15, respectively, was observed in a spiral microchannel with a U-turn on the way of particle motion in this investigation.

- Simulation results demonstrated that the WBCs, and CTCs focusing can be possible at Q = 1.7 mL/min due to the maximum distances between the WBCs, and CTCs. At this golden flow rate about 93% and 92% of the CTCs and the WBCs were isolated from the mixture, respectively. The performance of the examined chip indicated a perfect agreement with the simulation results and theoretical principles.
- Unlike other inertial focusing methods, traversing inner loops to outer loops by employing a high curvature ratio turn (U-turn) in a spiral pattern enhances the focusing behavior of both CTCs and WBCs, resulting in high purity with significantly high throughput.
- A size-based particle separation was performed and improved in this test at a high-throughput of about 1.7 mL/min, which gives an approximately twofold flow rate compared with other similar microchips.
- Comparison between the equilibrium position of cells and standard particles illustrated that the stiffness and elasticity effect should be considered in future simulations.

Recent advancements in inertial microfluidics provide a vast range of applications. According to the results of this research, it is possible to separate the CTCs from the patient's bloodstream using an unconventional spiral microchannel with high throughput. However, more studies must be performed to bring this chip into clinical circumstances.

## Supplementary Information

Supplementary Legends.

Supplementary Video 1.

Supplementary Video 2.

Supplementary Video 3.

## Data Availability

All data generated or analyzed during this study are included in this article, other additional datasets used during the current study is available from the corresponding author on reasonable request.
