# Supplementary material for: High-throughput isolation of cancer cells in spiral microchannel by changing the direction, magnitude and location of the maximum velocity
Source: Sci Rep. 2023 Feb 24;13:3213. doi: 10.1038/s41598-023-30275-x (PMC9958115; doi:10.1038/s41598-023-30275-x)
Supplement: Supplementary file 1 — Supplementary Legends. [file 41598_2023_30275_MOESM1_ESM.docx]

**Supplementary information**

Movie V1: This video shows the outlet of large particles. As can be seen, there are very few small particles in it. For this purpose, the sample is swept in all directions with a microscope.

Movie V2: This video shows the outlet of small particles, and as can be seen, there are very few large particles in it. For this purpose, the sample is swept in all directions with a microscope.

Movie V3: This video was taken online from the expansion before the outlets. As can be seen, by increasing the fluid flow rate to 1.7 ml/min, a focused line of larger particles is created, and almost all the larger particles go out of the desired outlet.
